# NOL6 Regulates the Proliferation and Apoptosis of Gastric Cancer Cells *via* Regulating TP53I3, CDK4 and MCM7 Expression

**DOI:** 10.3389/fonc.2022.708081

**Published:** 2022-04-12

**Authors:** Lei He, Xiaohan Qian, Pingping Ge, Dong Fan, Xiang Ma, Qiong Wu, Jin Sun, Lihua Yang, Jian Shen, Lijian Xu

**Affiliations:** ^1^ Department of General Surgery, The Second Affiliated Hospital of Nanjing Medical University, Nanjing, China; ^2^ Medical Center for Digestive Disease, The Second Affiliated Hospital of Nanjing Medical University, Nanjing, China

**Keywords:** NOL6, gastric cancer, proliferation, apoptosis, TP53I

## Abstract

**Background:**

Gastric cancer (GC) is a prevalent cancer with high mortality and strong invasiveness, and the entire regulatory networks of GC is still unclear.

**Objective:**

The aim of this study was to explore the specific mechanism of the effect of nucleolar protein 6 (NOL6) on the proliferation and apoptosis of GC cells.

**Methods:**

The human gastric adenocarcinoma cell line HGC-27 and AGS were cultured. qRT-PCR was used to verify the expression level of NOL6 in GC cells; MTT and EdU were used to test cell proliferation; TUNEL staining and Flow cytometry were used to detect cell apoptosis; The downstream genes and pathways following NOL6 knockdown were explored through the microarray assay and ingenuity pathway analysis, and the downstream genes were finally verified by qRT-PCR and Western blotting. The xenograft mice were used to investigate the effect of NOL6 on GC *in vivo*.

**Results:**

TCGA data analysis showed that NOL6 expression level was higher in GC cells than adjacent normal cells. Over-expression of NOL6 increased proliferation and colony formation, and inhibited the apoptotic rate in AGS and HGC-27 cells, while NOL6 knockdown induced the opposite effects. Through microarray assay and IPA analysis, NOL6-related downstream genes and critical signaling pathways were found. And we verified the relationship between downstream genes and GC. Additionally, NOL6 knockdown could decrease the weight and volume of tumor in the mice.

**Conclusion:**

NOL6 knockdown could inhibit cell proliferation and induce cell apoptosis of GC, suggesting that NOL6 may serve as a potential therapeutic target for treating GC.

## Introduction

Gastric cancer(GC)is a highly malignant tumor of the digestive system (1, 2). GC is the fifth most common cancer and the third leading cause of cancer death worldwide, with a 30% overall five-year survival rate (3–5). Adenocarcinoma is the most common pathological type of GC, accounting for more than 95% of all malignant cases (3, 6). Previous studies demonstrated that treatment of GC at early stage was effective, with 5-year survival rate of 90% (7, 8). However, most of the diagnosed patients are already in advanced stages of the disease, with lymphatic and distant metastasis, and the prognosis and postoperative survival are poor (9). Therefore, the molecular mechanism and potential biomarkers of GC must be further explored, providing effective targets for treating GC (10, 11).

In recent years, the pathogenesis and molecular mechanism of GC have been further studied. Although many genes (K-ras, HER2, PTK2, PIK3CA) have been confirmed to be involved in the development of GC, the gene networks of GC are still not thoroughly studied and many genes have not been elucidated (12–15). NOL6 is located at chromosome 9p13, spanned 11434 bps, and it is consisted of 26 relatively short exons and 25 introns (16). NOL6 is known as a protein coding gene, which encodes a nucleolar RNA-associated protein (NRAP) (16, 17). Nucleolus is the site of rRNA gene storage, rRNA synthesis processing, and assembly of ribosomal subunits (18, 19). Current studies indicated that NRAP was distributed on the inner surface of the chromosome and was involved in the transport of rRNA transcripts during mitosis in ribosomal organisms (16, 20, 21). Previously, only one study reported an abnormally high expression of NOL6 in prostate cancer, and overexpression of NOL6 promotes proliferation and inhibits apoptosis of prostate cancer cell (22). Although the role of NRAP in cell mitosis is strongly validated, the mechanism by which NOL6 gene acts on cell remains unclear.

Therefore, in this study, we explored the roles of NOL6 on the GC *in vitro* and *in vivo*. We hypothesized that NOL6 may be an oncogene in GC. Knockdown of NOL6 could inhibit the proliferation and promote apoptosis in GC. And the specific mechanism was explored by the gene sequencing and cell/molecular biology methods.

## Results

### NOL6 Gene Was Highly Expressed in Gastric Cancer Cells

**Figure 1:**
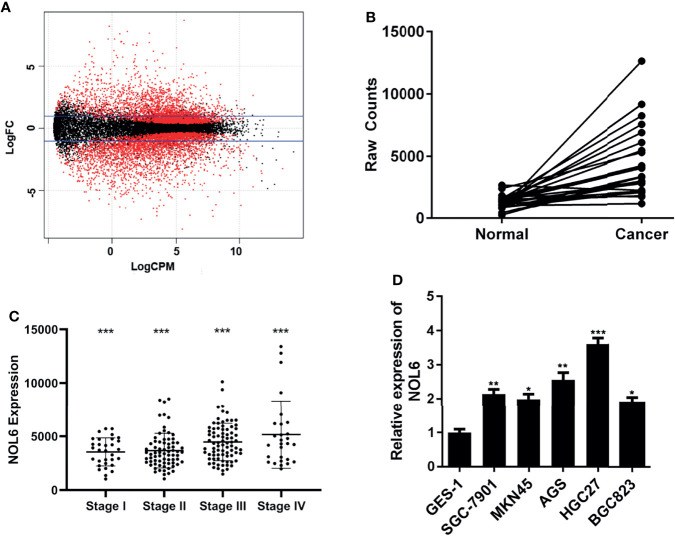
NOL6 gene was highly expressed in gastric cancer cells. **(A)** Differentially expressed genes generated by DEGseq package in ‘R’. The red points indicated the statistically significant differentially expressed genes in gastric cancer; logFC: log-fold-changes between each pair of RNA samples; logCPM: log2 counts-per-million. **(B)** NOL6 expression was observed obviously higher in gastric cancer tissues than that in adjacent normal tissues. **(C)** The NOL6 expression in the tissues from different clinical pathological stage was evaluated by qRT-PCR. **(D)** The gene expression of NOL6 in six cell lines (GES-1, SGC-7901, MKN45, HGC-27, BGC-823 and AGS) 7(*p < 0.05, **p < 0.01, ***p < 0.001).

At the beginning of the study, we downloaded gastric cancer data from TCGA database to explore differentially expressed genes in gastric cancer. After processing and analyzing the downloaded RNAseq and RNAseqv2 paired sample data, we obtained the differentially expressed genes in gastric cancer (**Figure 1A**). Candidate genes were then further screened and randomly condensed before NOL6 was finally selected as the target gene for further analysis. Results suggested that NOL6 was highly expressed in tumor tissue compare to para-tumor tissue (**Figure 1B**). Then, we compared the expression of NOL6 in gastric cancer at different pathological stages, and found that the TNM stage of gastric cancer was correlated with the expression of NOL6 (**Figure 1C**). Finally, survival analysis showed that the overall survival (OS) of gastric cancer patients with NOL6 overexpression was worse than that of the control group (**Figure 1D**).

### NOL6 Gene Was Highly Expressed in AGS and HGC-27 Cells

In order to accurately explore the role of the NOL6 gene in the development of gastric cancer, we require the cell line with a higher level of NOL6 gene expression to perform the experiment. The expression of NOL6 gene in six cell lines (GES-1, SGC-7901, MKN45, HGC-27, BGC-823 and AGS) was measured by qRT-PCR, and the results suggested that the gene expression in AGS and HGC-27 was higher than others, indicating that the AGS and HGC-27 cell lines were ideal cell models for further experiments.

### Over-Expression of NOL6 Increased Proliferation and Colony Formation of AGS and HGC-27 Cells

**Figure 2:**
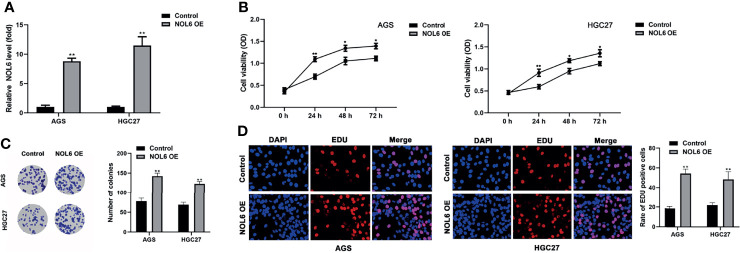
Over-expression of NOL6 increased proliferation and colony formation of AGS and HGC cells. **(A)** Effect of NOL6 OE on the gene expression of NOL6 in the AGS and HGC cells. **(B)** Effect of NOL6 OE on the viability of the AGS and HGC cells was evaluated by MTT assay. **(C)** Effect of NOL6 OE on the number of colonies in the AGS and HGC cells was detected by colony formation assay. **(D)** Effect of NOL6 OE on the proliferation of the AGS and HGC cells was assessed by EDU staining. (*p < 0.05, **p < 0.01, v.s.si-nc).

Next, we designed NOL6 over-expression plasmid to over- express NOL6 in AGS and HGC-27. The qRT-PCR results showed that the mRNA levels in NOL6 OE transfected cells were significantly higher than control cells (**Figure 2A**). And MTT, EdU and colony formation assay were used to detect the viability and proliferation of the AGS and HGC-27 cells. The results showed that the viability and proliferation were significantly increased in the AGS and HGC-27 cells of NOL6 OE transfected group (**Figures 2B, D**). In addition, the number of colony in NOL6 OE transfected group was markedly increased compared with control group (**Figure 2C**).

### Over-Expression of Inhibited the Apoptosis of AGS and HGC-27 Cells

**Figure 3:**
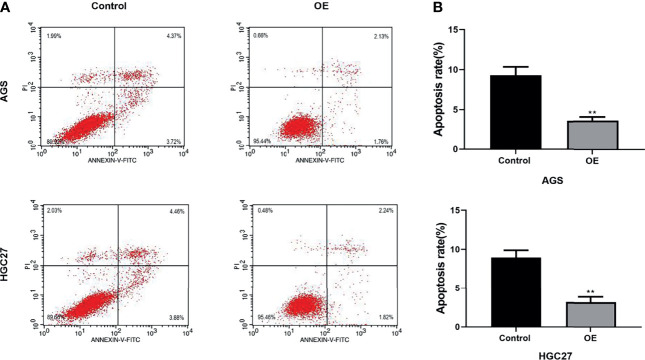
Over-expression of NOL6 decreased the apoptosis of AGS and HGC cells. **(A, B)** Flow cytometry was performed to measure the apoptosis rate of AGS and HGC cells. (**p < 0.01 v.s.si-nc).

Flow cytometry has been applied to evaluate cell apoptosis after NOL6 over-expression. Flow cytometry confirmed that the apoptosis rate was lower in NOL6 OE transfected group than that in the control group (**Figures 3A, B**).

### NOL6 Knockdown Suppressed Proliferation and Colony Formation of AGS and HGC-27 Cells

**Figure 4:**
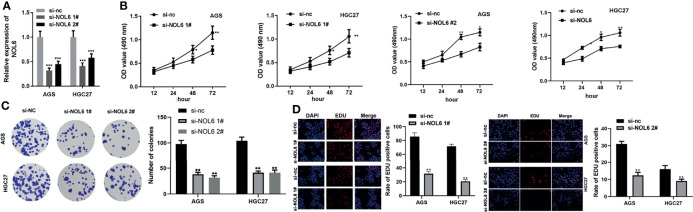
NOL6 knockdown suppressed proliferation and colony formation of AGS and HGC cells. **(A)** Effect of si-NOL6 1# and si-NOL6 2# on the gene expression of NOL6 in the AGS and HGC cells. **(B)** Effect of si-NOL6 on the viability of the AGS and HGC cells was evaluated by MTT assay. **(C)** Effect of si-NOL6 on the number of colonies in the AGS and HGC cells was detected by colony formation assay. **(D)** Effect of si-NOL6 on the proliferation of the AGS and HGC cells was assessed by EDU staining. (*p < 0.05, **p < 0.01, ***p < 0.001 v.s.si-nc).

Next, we designed si-NOL6 to knock down the expression of NOL6 in AGS and HGC-27 with si-nc as control. The qRT-PCR results showed that the mRNA levels in the si-NOL6 1# and si-NOL6 2# transfected cells were significantly lower than those in the si-nc transfected cells (**Figure 4A**). And MTT, EdU and colony formation assay were used to detect the viability and proliferation of the AGS and HGC-27 cells. The results showed that the viability and proliferation were significantly decreased in the AGS and HGC-27 cells of si-NOL6 transfected group (**Figures 4B**, **D**). In addition, the number of colony in si-NOL6 transfected group was remarkable increased compared with the si-nc transfected group (**Figure 4C**).

### NOL6 Knockdown Promoted the Apoptosis of AGS and HGC-27 Cells

**Figure 5:**
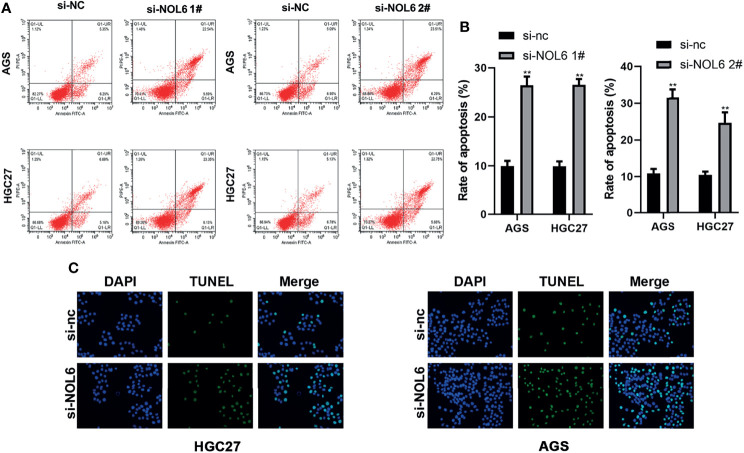
NOL6 knockdown promoted the apoptosis of AGS and HGC cells. **(A, B)** Flow cytometry was performed to measure the apoptosis rate of AGS and HGC cells. **(C)** The apoptosis of AGS and HGC cells by the TUNEL staining. (**p < 0.01, v.s.si-nc).

The TUNEL staining and flow cytometry were applied to evaluate cell apoptosis after NOL6 knockdown. Flow cytometry confirmed that the apoptosis rate was higher in si-NOL6 transfected group than that in the si-nc transfected group (**Figures 5A, B**). TUNEL staining also showed that the number of apoptotic cells in si-NOL6 group was significantly increased compared with the si-nc group. (**Figure 5C**).

### NOL6-Releated Critical Pathways Involved in Tumor Progression

**Figure 6:**
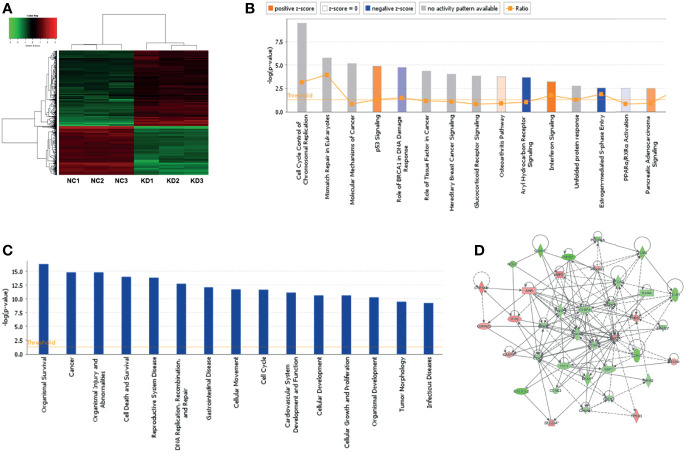
Microarray assay and IPA analysis following NOL6 knockdown. **(A)** The clusters diagram showed the differentially expressed genes after NOL6 inhibited. The red plots indicated the up-regulated genes, while the green plots indicated the down-regulated genes. **(B)** Top 15 classical signaling pathways following NOL6 knockdown were list in the canonical pathway histogram. All signaling pathways were sorted by –Log(P-value), the orange histogram represented apparently activated and the blue histogram represented apparently inhibited. **(C)** Gene enrichment of disease and function following NOL6 knockdown. All diseases and functions are sorted by -log (p-value). **(D)** The gene interaction network shows the interaction between downstream molecules after NOL6 silencing. The red plots represented up-regulated genes, the green plots represented down-regulated genes, and the intensity of the color indicates degree of variation. Solid lines represent direct interaction, whereas dotted line represent indirect interaction.

Microarray assay was carried out to detect downstream genes after NOL6 knockdown, and 978 differentially expressed genes were detected in the NOL6 knockdown group, among which 581 were up-regulated and 397 were down-regulated (|Fold Change| >1.5, P-value<0.05). (**Figure 6A**). The downstream critical pathways were analyzed by ingenuity pathway analysis (IPA). The enrichment of differential genes in classical signaling pathways were shown in the canonical pathway histogram and the top 15 pathways were listed, among which Aryl Hydrocarbon Receptor (AHR) Signaling was the most significantly inhibited and Interferon Signaling was the most obviously activated pathway (**Figure 6B**). The enrichment of differential genes in disease and function classification was shown in the disease and function histogram (**Figure 6C**). The gene interaction network showed the interaction between downstream molecules after NOL6 expression silencing (**Figure 6A**). TP53I3, CDK4, PARP1, CDK2 CHEK1 and MCM7 were analyzed and deemed as downstream genes, which was associated with the gene interaction network following NOL6 knockdown (**Figure 6D**). These genes were selected for further analysis.

### NOL6 Knockdown Up-Regulated TP53I3 Expression and Down-Regulated CDK4 and MCM7 Expression

**Figure 7:**
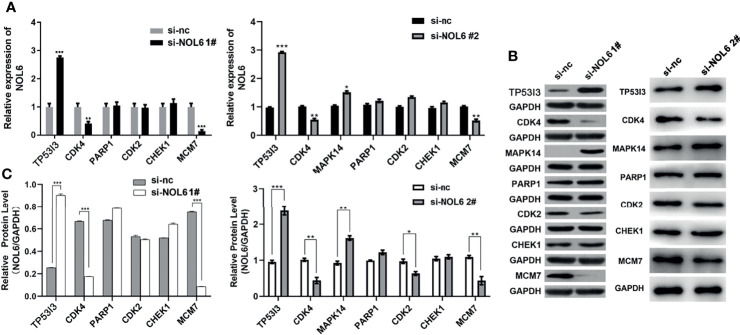
Expression changes of downstream genes following NOL6 knockdown. **(A)** Effect of si-NOL6 on the gene expression of TP53I3, CDK4, PARP1, CDK2, CHEK1 and MCM7 in the AGS cells was detected by qRT-PCR. **(B, C)** Effect of si-NOL6 on the protein expression of TP53I3, CDK4, PARP1, CDK2, CHEK1 and MCM7 in the AGS cells was evaluated by western blot. (*p < 0.05, **p < 0.01, ***p < 0.001 v.s. si-nc).

The expression levels of TP53I3, CDK4 and MCM7 were determined by qRT-PCR and western blotting assay. The qRT-PCR results illustrated that TP53I3 mRNA expression was up-regulated and the CDK4 and MCM7 mRNA expressions were down-regulated following NOL6 knockdown (**Figure 7A**). And western blotting experiment also confirmed that TP53I3 was up-regulated and CDK, MCM7 were significantly down-regulated on protein following NOL6 knockdown (**Figures 7B, C**).

### OE-CDK4, OE-MCM7 and si-TP53I3 Could Reverse the Effect of NOL6 on Proliferation and Apoptosis of AGS and HGC-27 Cells

**Figure 8:**
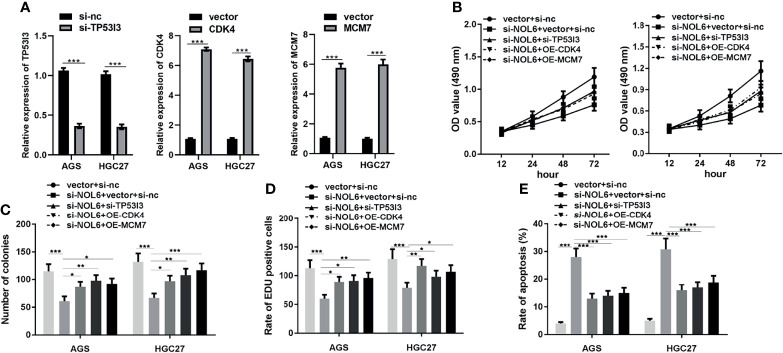
Effect of CDK4, MCM7 and si-TP53I3 on proliferation and apoptosis in NOL6 knockdown AGS and HGC-27 cells. **(A)** Effect of CDK4, MCM7 and si-TP53I3 transfection on the gene expression of CDK4, MCM7 and TP53I3 in the AGS and HGC-27 cells. **(B)** Effect of CDK4, MCM7 and si-TP53I3 on the viability of the NOL6 knockdown AGS and HGC cells. **(C)** Effect of CDK4, MCM7 and si-TP53I3 on the number of colonies in the NOL6 knockdown AGS and HGC cells. **(D)** Effect of CDK4, MCM7 and si-TP53I3 on the proliferation of the NOL6 knockdown AGS and HGC cells. **(E)** Effect of CDK4, MCM7 and si-TP53I3 on the apoptosis of the NOL6 knockdown AGS and HGC cells. (*p < 0.05, **p < 0.01, ***p < 0.001).

Moreover, we designed OE-CDK4 and OE-MCM7 to promote the expression of CDK4 and MCM7 in AGS and HGC-27 cells with vector as control. And we also designed si-TP53I3 to inhibit the expression of TP53I3 with si-nc as control. The qRT-PCR results confirmed that OE-CDK4 and OE-MCM7 transfection could effectively promote the expression of CDK4 and MCM7 genes, and si-TP53I3 transfection could effectively inhibit the expression of TP53I3 gene in AGS and HGC-27 cells (**Figure 8A**).

Then we measured the effects of OE-CDK4, OE-MCM7 and si-TP53I3 on the proliferation and apoptosis in AGS and HGC-27 cells. The MTT, EdU and colony formation assay showed that OE-CDK4, OE-MCM7 and si-TP53I3 could significantly increase the proliferation and inhibited the apoptosis in AGS and HGC-27 cells (**Figures 8B–E**).

### NOL6 Silencing Could Inhibit the Tumor Growth of GC *In Vivo*

**Figure 9:**
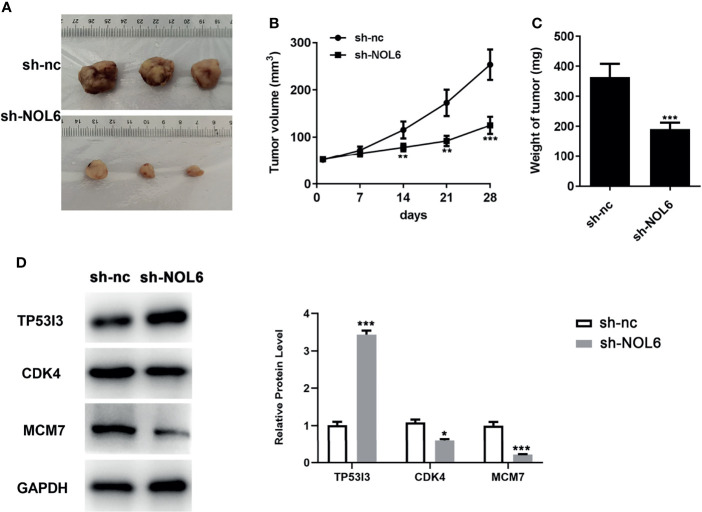
Effect of NOL6 silencing on the growth of tumor in the nude mice. **(A)** The image of the tumor. **(B)** Effect of sh-NOL6 on the volume of the tumors in the mice. **(C)** Effect of NOL6 silencing on the weight of the tumors in the mice. **(D)** Expressions of TP53I3, CDK4 and MCM7 in different groups (*p < 0.05, **p < 0.01, ***p < 0.001 v.s. sh-nc.).

Finally, we further conducted animal experiments to explore the effect of NOL6 inhibition on tumor growth. Results showed that inhibition of NOL6 can significantly decrease the volume and weight of the tumor compared with the sh-nc group (**Figures 9A–C**). Moreover, knockdown of NOL6 increased the expression of TP53I3, and decreased the expression of CDK4 and MCM7(**Figure 9D**).

## Discussion

Tumor biogenesis is a complex progress involving multiple genes, and HOXC10 (22), UFM1 (23), EGR1 (24), MDGA2 (25), and CLDN18 (26) have been proved to be biomarkers of GC, but the pathogenesis and mechanism of GC have not been clarified. It is necessary for us to further study the pathogenesis and mechanism of GC in order to obtain new ideas for diagnosis and treatment of GC.

Previous reports had shown that increased ribosomes biogenesis could regulate the mRNA and protein biogenesis and promote cell growth and tumor biogenesis (27). Benjamin Albert et al. further proved that NOL6, a nucleolar protein gene, stimulated cell growth and colony formation by regulating Ifh1 (28). However, the mechanism of NOL6 in promoting cell growth and tumor biogenesis had not been clarified. This was the first time that NOL6 had been identified as a regulatory factor in the development of GC. In this study, we downloaded the RNAseq and RNAseqv2 paired sample data from TCGA database, and found that NOL6 expression in GC cells was higher than that in adjacent tissues, and the pathological TNM stage was proved to be significantly relate to the expression of NOL6. The results suggested that NOL6 was involved in the progression of GC. Moreover, NOL6 over-expression could increase cell proliferation, colony formation, and inhibit cell apoptosis, while NOL6 knockdown could suppress cell proliferation, decrease the ability of colony formation, and induce cell apoptosis. What’s more, we found that the weight and volume of the tumor in the si-NOL6 mice were significantly decreased, which was consist with our results *in vitro*. Therefore, we believed that NOL6 was closely related to the occurrence and development of GC.

With the development of technology, sequencing can analyze the relationship between genes and diseases from a macro perspective. In this study, microarray and IPA analysis were conducted to explore the possible downstream targets after NOL6 was knocked down, which suggested that TP53I3, CDK4 and MCM7 were involved in the NOL6 network. The results of qRT-PCR and WB showed that after NOL6 inhibition, the expression of TP53I3 was up-regulated, and the expression of CDK4 and MCM7 was down-regulated, which was not only consistent with the results of microarray assay and IPA, but also confirm their reliability. CDK4, a member of the d-type cyclin family, which can interacted with AHR to form a complex and has been implicated in the phosphorylation of retinoblastoma gene product (Rb) and plays an important role in the G1 to S phase progression of the cell cycle (29, 30). In addition, CDK4 has been shown to participate in processes outside the regulation of the cell cycle leading to tumor development, including cell senescence, gene repair and metabolism (31). Currently, the CDK4 inhibitor Palbociclib has been used in the clinical treatment of breast cancer with a longer progression-free survival (PFS) than that in the control group (32). In addition, studies has shown that genes such as RN181, KLF16 and PCAF were involved in the progress of gastric cancer by regulating CDK4 levels (33–35). MCM7, as a highly conserved protein in eukaryotes, belongs to the minichromosome maintenance (MCM) protein family and shares sequence homology with MCM2-6, which contains DNA helicase activity and involved in the core process of DNA replication (36). The Rb fragment can specifically bind to MCM7 to inhibit the ATPase activity of MCM7, leading to the inhibition of DNA replication and blocking cell cycle in G1 phase, whereas CDK4 can also react with MCM7 to dissociate the Rb and MCM7 complex and promote the cell cycle into S phase (37). It has been reported that MCM7 is highly expressed in hepatocellular carcinoma, prostate cancer, esophageal cancer, lung cancer, etc. (38–41). Low MCM7 level can inhibit the proliferation and invasion of gastric adenocarcinoma cells, and high expression of MCM7 is associated with short survival, which can effectively predict prognosis, indicating that MCM7 is a potential biomarker and target for gastric adenocarcinoma (42). TP53I3, also known as the tumor protein p53 inducible protein 3, is encoded by the downstream gene of P53 and is thought to participate in the p53 mediated transcription process (43). The association between TP53I3 and P53 is that TP53I3 promote the ubiquitylation of p53 and affect the level of p53 protein, TP53I3 knockdown could accelerate the degradation of p53 and inhibit cells apoptosis (44). Studies had found that the expression of TP53I3 was generally decreased in gastric cancer tissues, and the up-regulation of TP53I3 inhibited the proliferation of tumor cells and induced apoptosis (45). The results had revealed that NOL6 knockdown affect CDK4, MCM7 and TP53I3 expression. On the other hand, TP53I3 knockdown, CDK4 and MCM7 over-expression also can reverse the effect of si-NOL6 on the proliferation and apoptosis in GC. These results further confirmed that NOL6 regulates the proliferation and apoptosis of gastric cancer cells *via* regulating TP53I3, CDK4 and MCM7.

## Conclusion

In summary, we first demonstrated that NOL6 can regulate the oncogenic behaviors of gastric cancer cells by down-regulating TP53I3 and up-regulating CDK4, MCM7. The results of current work indicated that NOL6 may become a new therapeutic target for the treatment of GC and provide basis for the further development of new targeting drugs for GC.

## Methods and Materials

### The Cancer Genome Atlas (TCGA) and Survival Analysis

TCGA database was used to obtain the differential expressed genes and the filter was used to reveal and download the desired dataset. The cancer type was set to stomach, the program was set to TCGA-STAD, and the data type was set to RNA-Seq. Among the 443 available data samples, 32 pairs RNAseq and RNAseqv2 paired samples with pathological information were selected and analyzed for the next experiment. For the gene symbol that reads >50, the Trimmed Mean of M-values (TMM) was used for data normalization and the biological coefficient of variation (BCV) was used for quality control. Then the differential expressed genes were further screened by the following criteria: i) Remove genes that have been reported to have functional and clinical relevance; ii) Remove transmembrane protein genes; iii) Remove genes with unclear annotation; iv) Remove genes with >100 papers in Pubmed; v) Remove genes that have had previous results. In order to clearly explore the relationship between NOL6 expression and GC, we analyzed the expression levels of NOL6 in tumor tissues and adjacent tissues, and explored the differences in the expression of NOL6 in different pathological stages through data downloaded from TCGA. KM Plot, an online survival analysis website (https://kmplot.com/), was used to further analyze the NOL6 expression level and the survival of gastric cancer patients. The sequencing data were uploaded to GEO database(https://www.ncbi.nlm.nih.gov/geo/query/acc.cgi?acc=GSE193606).

### Cell Culture

The human gastric adenocarcinoma cell line HGC-27 and AGS were purchased from the Cell Bank of the Chinese Academy of Sciences (Shanghai, China). The cells were cultured in DMEM medium containing fetal bovine serum at 37°C in an atmosphere of 5% CO_2_.

### Real-Time Quantitative PCR (qRT-PCR)

The TRIzol(Shanghai Pufei Biotech Co., Ltd)was used to extract and purify the total RNA from cells according to the manufacture’s instruction, the cDNA was synthesized by using the MMLV kit (Promega). The NOL6 primers (forward primer:TGAGGCACGGCTGTCTATGAT, reverse primer: GGAGATGCAGGACATGGTC) was purchased from Guangzhou RiboBio Co.,LTD, and GAPDH (forward primer: TGACTTCAACAGCGACACCCA, reverse primer: CACCCTGTTGCTGTAGCCAAA) was set as the internal control group. The gene expression was detected by using the SYBR Master Mixture Kit (TAKARA) in Real-Time PCR platform LightCycler480 (Roche), and the reaction conditions were: 95°C for 30s and followed by 40 cycles of 95°C for 5s, 60°C for 30s and dissociation at 95°C for 15s, 60°C for 30, 95°C for 15s. The relative gene expression level (NOL6/GAPDH) in experimental cells and control cells was compared and the data was finally processed using the 2-ΔΔCt method. The NOL6 expression in GES-1, SGC-7901, MKN45, HGC-27, BGC-823 and AGS was detected using qRT-PCR to pick the ideal cell line with a higher level of NOL6 for the next experiment. And the expression level of the target gene in si-NOL6 and si-nc transfected cells and changes of downstream genes TP53I3 (Forward Primer: GGAGGACCGGAAAACCTCTAC, Reverse Primer: CCTCAAGTCCCAAAATGTTGCT), CDK4 (forward primer: GGGGACCTAGAGCAACTTACT, reverse primer: CAGCGCAGTCCTTCCAAAT), MCM7 (forward primer: CCTACCAGCCGATCCAGTCT, reverse primer: CCTCCTGAGCGGTTGGTTT), PARP1 (forward primer: ACAGCCTGTACCACCCTCC, reverse primer: AGCACCTGGTGATTTGGCATC) and CDK2 (forward primer: TGTTTAACGACTTTGGACCGC, reverse primer: CCATCTCCTCTATGACTGACAGC) after NOL6 knockdown were also verified by qRT-PCR.

### Cell Transfection

NOL6 over-expression plasmids (NOL6 OE), Negative control si-nc, vector and NOL6 knockdown plasmids (si-NOL6), TP53I3 knockdown plasmids (si-TP53I3), CDK4 overexpression plasmids (OE-CDK4) and MCM7 overexpression plasmids (OE-MCM7) were purchased from Shanghai GenePharma Co., Ltd. (Shanghai, China). The AGS and HGC-27 cells were cultured in F12+10%FBS medium for 24h until the cell confluence reached 80%.Then the cells in logarithmic growth phase were chosen to be digested by pancreatin, inoculated into 6-well culture plates and added with appropriate amount of virus for infection. After transfection, the cells were further cultured 72 h for further analysis.

### Western Blot

After washing cells with PBS solution twice, the cells were lysed with RIPA buffer (Beyotime Biotechnology) for 20 min to get total protein, and the concentration was adjusted to 2 μg/μl using BCA Protein Assay Kit (Beyotime Biotechnology). The proteins were separated by 10% SDS-PAGE solution and were transferred to the PVDF membrane (Millipore) by transfer electrophoresis at 4°C in TBST overnight. Then the PVDF membrane was incubated with the rabbit anti-NOL6 polyclonal antibody (1:400 dilution; no. ab50875; Abcam Trading Co. Ltd., Shanghai) at 4°C for 2h., and washed with TBST for 4 time (8min/time). The incubation product was further incubated with secondary antibody (Goat anti-mouse IgG-HRP:1:2000 dilution; no. sc-2005; Santa-Cruz Biotechnology) for 1.5h, and washed with TBST for 4 time (8 min/time). The condition of target proteins on the membrane were detected by X-ray after reaction with Pierce™ ECL Western Blotting Substrate (Thermo Fisher Scientific Inc.) in darkness for 5min. Finally, the quantitative detection of western blot was carried out through ImageJ (version 1.48V)

### MTT Assay

Logarithmic phase AGS and HGC-27 cells were treated with trypsin, and resuspended in complete medium to obtain cell suspensions, then the cells were seeded into a 96-well plate and cultured at 37˚C in an atmosphere of 5% CO_2_, with the cell concentration adjusted to 2000 cells/well. Since the day after inoculation, 20 μL 5 mg/mL MTT (Genview) was added to each well for 4h, then the supernatant was completely aspirated and discarded, then 100μL DMSO (Shanghai Shiyi Chemicals Reagent Co., Ltd., Shanghai, China) was added to each well and oscillate for 2-5 min. An enzyme mark instrument (Tecan infinite) was used to measure the optical density (OD) at 490 nm. Every experiment repeated three times.

### Colony Formation Assay

After lentivirus transfection, logarithmic phase AGS and HGC-27 cells were re-suspended into cell suspension and counted. The transfected cells were inoculated in the 6-well plate with the concentration of 1000 cells/well, and 3 multiple holes were set for each experiment. The inoculated cells were further cultured in the incubator at 37°C, 5% CO_2_ for 2 weeks, during which the culture medium was changed and the cell status was observed every 3 days. After 14 days, the cell colonies were photographed with fluorescence microscope (Olympus). Then the PBS were used to wash the cells once, 4% paraformaldehyde were added to fix cells, the GIEMSA were used to stain the cells, and the cells were washed with ddH_2_O for several times and were dried in the final. Condition of colony formation was photographed and the colony number was counted for each group.

### 5-Ethynyl-2-Deoxyuridine (EdU) Assay

Logarithmic phase AGS and HGC-27 cells were treated with trypsin, and resuspended in complete medium to obtain cell suspensions, then the cells were seeded into a 96-well plate and cultured at 37°C in an atmosphere of 5% CO_2_, with the cell concentration adjusted to 2000 cells/well. The proliferation of the cells were determined using a Cell-Light EdU DNA Cell Proliferation Kit (RiboBio, Guangzhou, China) according to the instructions of the manufacturer.

### Annexin V-APC Single Staining Flow Cytometry

Flow cytometry was performed to detect cell apoptosis. The transfected cells were cultured on a 6-well plate until the cells covered more than 70% of the well, then apoptosis kit was used to detect apoptosis. Briefly, the cells were digested with trypsin to obtain the cell suspension, and collected with the cell supernatant in a 5ml centrifuge tube. After centrifuging for 5 min, precipitated cells were collected and centrifugated again for 3 min. Then 200 uL 1×binding buffer and 10 um Annexin V-APC staining was added, and the cell apoptosis was detected by flow cytometry (Millipore) after 15 min at room temperature in dark.

### TUNEL Staining

Apoptosis of AGS and HGC-27 cells were observed by TUNEL staining. The transfected cells were cultured on a 6-well plate until the cells covered more than 70% of the well. Then discarded the culture medium. The cells were fixed with 4% formaldehyde for 15 min and dehydrated with 50%, 75%, 95% and 100% ethanol for 5 min. Then washed twice with phosphate buffer solution and treated with 0.5% Triton X-100 for 20 min. At last, added TUNEL working solution and incubated at 37°C for 1 h. The apoptotic cells were detected by fluorescence microscope

### Microarray Assay Analysis

Downstream gene effects after NOL6 knockdown were analyzed by GeneChip PrimeView Human Gene Expression Array (Affymetrix; No. 901838; Thermo6). Trizol reagents was used to extract the total RNA, the NanoDrop 2000 (Thermo) and Agilent 2100 Bioanalyzer (Aglient) were used to evaluate and control the total RNA quality, and the qualified samples were subjected to microarray assay. The GeneChip 3’IVT Express Kit (Affymetrix) was employ for the preparation of amplified RNA (aRNA), including: single DNA template synthesis, double-stranded DNA template synthesis and reverse transcription *in vitro*. GeneChip Hybridization Oven 645 (Affymetrix) was applied to hybridize fragmented aRNA and GeneChip and GeneChip Fluidics Station 450 (Affymetrix) was used for gene washing. At last, GeneChip Scanner 3000 was used to scan the chip array to gain raw data and images. Genes with significant differences after NOL6 knockdown were selected from the chip analysis results: |Fold Change| must be greater than 1.5, and the p-value should be less than 0.05.

### Ingenuity Pathway Analysis (IPA)

IPA was used to analyze the dataset of differential expressed genes obtained from the gene microarray chip, including the chip probe number and the Fold Chang value. Analysis of ‘Classic Pathway’ and ‘Disease and Function’ following NOL6 knockdown were performed based on multiple public databases through IPA. The enrichment of Pathway and Disease and Function Classification were sorted by -log (p-value) and the activation or inhibition of the molecule was represented by Z-score. The gene interaction network was also analyzed using IPA.

### Animal Experiments

The nude BALC mice (18 ± 2g) were purchased from Animal center of Nanjing Medical University. The AGS cells were cultured in medium for 24h until the cell confluence reached 80%. The cells in logarithmic growth phase were chosen to be digested by pancreatin, inoculated into 6-well culture plates and added with sh-nc and sh-NOL6 for infection. The mice were randomly divided into sh-nc group and sh-NOL6 group, six in each group. The AGS cells (2×10^8^ cells/mL) were injected subcutaneously into the mice. Vernier caliper was used to measure the long diameter (L) and short diameter (S) of tumor once a week, and the volume (V) was calculated by the following formula: V=0.5×L×W^2^. Four weeks later, the mice were anesthetized by intraperitoneal injection of 3% pentobarbital (160 mg/kg) and killed. The tumor weight was measured.

### Statistical Analysis

The experimental data were demonstrated as mean ± standard deviation (SD), and the statistical significance of the differences between groups was examined by the Student’s t-test; expression of NOL6 and pathological stage of tumor was analyzed by Mann-Whitney U test. All data was processed by Microsoft Office Excel 2007, and charts were drawn with GraphPad prism (Version 8.0.2). P-value less than 0.05 were considered statistically significant.

## Data Availability Statement

The datasets presented in this study can be found in online repositories. The names of the repository/repositories and accession number(s) can be found in the article/supplementary material.

## Ethics Statement

The animal experiment was approved by the Ethics Committee of The Second Affiliated Hospital of Nanjing Medical University.

## Author Contributions

LH and PG design of the work. DF and XM the acquisition, analysis. QW and JSun interpretation of data. LX and JSun have drafted the work or substantively revised it. XQ and LY performed all the revisions and provided the funds. All authors read and approved the final manuscript.

## Conflict of Interest

The authors declare that the research was conducted in the absence of any commercial or financial relationships that could be construed as a potential conflict of interest.

## Publisher’s Note

All claims expressed in this article are solely those of the authors and do not necessarily represent those of their affiliated organizations, or those of the publisher, the editors and the reviewers. Any product that may be evaluated in this article, or claim that may be made by its manufacturer, is not guaranteed or endorsed by the publisher.

## Abbreviations

GC, Gastric cancer; NOL6, nucleolar protein 6; NRAP, nucleolar RNA-associated protein; OS, overall survival; PFS, progression-free survival; MCM, minichromosome maintenance; TCGA, The cancer genome atlas; TMM, Trimmed Mean of M-values; BCV, biological coefficient of variation; EdU, 5-ethynyl-2-deoxyuridine; IPA, Ingenuity Pathway Analysis; SD, standard deviation.
